# Mammary epithelium permeability during established lactation: associations with cytokine levels in human milk

**DOI:** 10.3389/fnut.2024.1258905

**Published:** 2024-02-13

**Authors:** Katie T. Kivlighan, Sallie S. Schneider, Eva P. Browne, Brian T. Pentecost, Douglas L. Anderton, Kathleen F. Arcaro

**Affiliations:** ^1^College of Nursing, University of New Mexico Health Sciences Center, Albuquerque, NM, United States; ^2^Pioneer Valley Life Sciences Institute, Baystate Medical Center, Springfield, MA, United States; ^3^Department of Veterinary and Animal Sciences, University of Massachusetts Amherst, Amherst, MA, United States; ^4^Department of Sociology, University of South Carolina, Columbia, SC, United States

**Keywords:** human milk, sodium, Na/K ratio, cytokine, growth factor, inflammation, mammary epithelium permeability, subclinical mastitis

## Abstract

**Objective:**

The cytokine profile of human milk may be a key indicator of mammary gland health and has been linked to infant nutrition, growth, and immune system development. The current study examines the extent to which mammary epithelium permeability (MEP) is associated with cytokine profiles during established lactation within a sample of US mothers.

**Methods:**

Participants were drawn from a previous study of human milk cytokines. The present analysis includes 162 participants (98 Black, 64 White) with infants ranging from 1 to 18 months of age. Levels of cytokines were determined previously. Here we measure milk sodium (Na) and potassium (K) levels with ion-selective probes. Two approaches were used to define *elevated MEP*: Na levels ≥10 mmol/L and Na/K ratios greater than 0.6. Associations between maternal–infant characteristics, elevated MEP, and twelve analytes (IL-6, IL-8, TNFα, IL-1β, FASL, VEGFD, FLT1, bFGF, PLGF, EGF, leptin, adiponectin) were examined using bivariate associations, principal components analysis, and multivariable logistic regression models.

**Results:**

Elevated MEP was observed in 12 and 15% of milk samples as defined by Na and Na/K cutoffs, respectively. The odds of experiencing elevated MEP (defined by Na ≥ 10 mmol/L) were higher among Black participants and declined with older infant age. All cytokines, except leptin, were positively correlated with either Na or the Na/K ratio. A pro-inflammatory factor (IL-6, IL-8, TNFα, IL-1β, EGF) and a tissue remodeling factor (FASL, VEGFD, FLT1, bFGF, PLGF, adiponectin) each contributed uniquely to raising the odds of elevated MEP as defined by either Na or the Na/K ratio.

**Conclusion:**

This exploratory analysis of MEP and cytokine levels during established lactation indicates that elevated MEP may be more common in US populations than previously appreciated and that individuals identifying as Black may have increased odds of experiencing elevated MEP based on current definitions. Research aimed at understanding the role of MEP in mammary gland health or infant growth and development should be prioritized.

## Graphical abstract

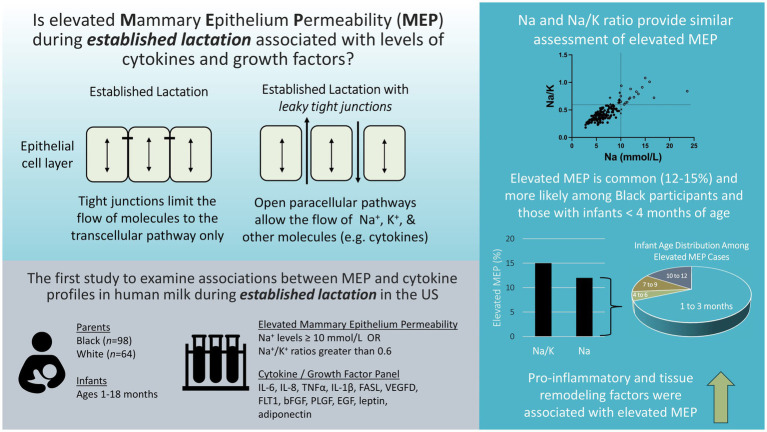

## 1 Introduction

Human milk is a complex biological fluid containing a multitude of cellular and molecular components integral to the health or disease state of the mother and infant. In particular, inflammatory markers in human milk appear to be associated with infant nutrition (1, 2), growth (3, 4), and immune system development (5, 6), and may be useful indicators of current (7, 8) and future mammary gland health (9, 10).

Human milk contains a variety of cytokines that act locally in the mammary gland and/or influence the growth and development of infant tissues (11). Cytokines represent a large class of secreted bioactive molecules that modulate cell-to-cell communication to impact cellular growth, viability, and differentiation, as well as immune and inflammatory responses (5). In human milk, cytokines are produced by leukocytes that have migrated into the mammary tissue from systemic circulation as well as by tissue-resident cells, such as mammary epithelium, fibroblasts, and adipocytes (5, 12). Specific cytokines are upregulated during mammary gland differentiation during pregnancy, in response to milk stasis, and during involution (13, 14). This upregulation is distinct from the rise in pro-inflammatory cytokines observed during an infection, such as mastitis. In the setting of infection, pro-inflammatory cytokines modulate the immune response of the mammary gland (7, 8).

Adipokines, such as leptin and adiponectin, are a subset of cytokines produced by adipose tissue that serve as endocrine signaling molecules with roles in regulating metabolism and body composition (11, 12). Both also play an important role in modulating mammary gland development and tissue remodeling during the lactation cycle (15). Growth factors are a class of cytokines linked to tissue growth and remodeling. They may retain bioactivity after ingestion and are important for the development of the infant intestinal barrier (11). Within the mammary gland, growth factors are important for angiogenesis, maintaining lactation, and regulating involution (16, 17).

Measurement of mammary epithelium permeability (MEP) may provide important information for the interpretation of cytokine concentrations in human milk. Prior to secretory activation (i.e., onset of mature milk), paracellular pathways between mammary epithelial cells are open allowing communication between the maternal bloodstream and the mammary gland (18). Following birth, rapid tight junction formation within the mammary epithelium facilitates paracellular pathway closure, a shift important for the establishment and maintenance of milk synthesis and secretion (19, 20). The paracellular pathway is particularly important for the movement of ions across the mammary epithelium. For example, sodium (Na) levels are high in colostrum, but decrease rapidly during the first 5 days postpartum in response to paracellular pathway closure. It has been proposed that once Na levels decrease below 10 mmol/L, milk maturity has been achieved (18, 21). In contrast, potassium (K) accumulates in milk as paracellular pathways close (18, 22). Both Na alone and as a ratio with potassium (Na/K) have been used to assess MEP. Na/K ratios of less than 0.6 have been used to indicate tight junction closure and milk maturity (18).

As long as the paracellular pathways remain closed during established lactation, milk secretion is maintained and levels of Na and the Na/K ratio remain low (19). However, both milk accumulation and inflammation have been linked to the re-opening of tight junctions and rising Na or Na/K ratios (19, 23). Our lab recently demonstrated that shifts in the Na/K ratio were closely linked to anti-SARS-CoV-2 antibody levels in human milk within a single individual over time, confirming an association between permeability and immune factors (24). Knowledge of MEP could provide important context for the interpretation of cytokine levels in human milk.

We previously examined levels of human milk cytokines in a cohort of Black and White participants during established lactation to determine associations with obesity, race, and risk factors for breast cancer (24). Of note, certain pro-inflammatory cytokines (IL-1β, FASL), growth factors (bFGF, EGF), and adipokines (leptin, adiponectin) were elevated among participants with a BMI >30. Levels of 1L-1β and leptin were found to be higher in Black participants.

The goals of the current study were (1) to determine the extent to which MEP as indicated by Na and Na/K ratios is associated with cytokine profiles in human milk during established lactation and (2) to determine if race or BMI are associated with elevated MEP. We hypothesized that higher levels of human milk pro-inflammatory cytokines would be associated with elevated permeability and that similar to observed patterns for the cytokines listed above, elevated MEP would be more common among participants with Black race or a BMI >30.

## 2 Methods

### 2.1 Study population

This is a secondary analysis of selected data from a study examining racial differences in cytokines in human milk in relation to breast cancer etiology (24). For the present study, we selected participants for whom archived whole milk samples were available. All participants had signed a consent form for a study approved by the Institutional Review Board of the University of Massachusetts Amherst (#749). Briefly, for the original study, all participants completed questionnaires on demographics and health history. Milk samples were collected between 2007 and 2013 from lactating females aged 18 years and older living in the continental United States (US). Participants had collected milk in the morning upon waking by expressing the full contents of each breast into separate glass or BPA-free plastic containers via hand expression or use of their own pump. Archived aliquots of whole milk used in this project had been stored at −20°C. Descriptive statistics regarding collection and storage and presented in Supplementary Table S1.

Of the 292 participants in the original study, frozen aliquots of whole milk were available from 167 participants (24). Of these, 5 mother-infant dyads were identified as being more than 3 SD above the mean for infant age (>798 days or 2.18 years of age). Breastfeeding practices employed by these participants were not typical of the remainder of the sample and were therefore excluded. Our final sample consisted of 162 participants (98 Black, 64 White) with infants ranging in age from 1 to 18 months.

### 2.2 Measurement of sodium and potassium ions

Levels of Na and K were measured using ion-selective electrode probes (Medica EasyLyte Na/K Analyzer) in 2022, providing new data for the current analysis. Briefly, 1 mL aliquots of whole milk were thawed, centrifuged at 3220 *g* for 3 min at 4°C and the Na and K concentrations (mmol/L) were determined in the clarified whey fraction. A total of 18 samples were run in duplicate with mean coefficients of variation (CV) of 7.8% for Na and 4.4% for K. A ratio was calculated between Na and K for each participant. Using Mann–Whitney *U* tests, neither Na nor the Na/K ratio were associated with prescription medication use, over-the-counter pain medication use, or shipping methods, *p*s < 0.10. Spearman rank correlations with years frozen at −20°C, and number of freeze–thaw cycle were also non-significant, *p*s < 0.10.

### 2.3 Measurement of cytokines and growth factors

Assays for pro-inflammatory cytokines, growth factors, and adipokines were performed in the original study (24). Briefly, multiplex and single-analyte electrochemical-luminescent sandwich assays from MesoScale Discovery (MSD, Gaithersburg, MD) were used to measure 15 analytes, of which 12 were selected for the present analysis and are shown in Table 1. IFN, TIE-2, and VEGFC were excluded due to low detectability (<35%). Thirty-eight samples, eight standards, and two of three controls were tested in duplicate on each plate according to the manufacturer’s protocols. The lower limit of detection (LLOD) for each included analyte was determined empirically, as previously reported in Murphy et al. (25), and is also reported in Table 1. Coefficients of variation (CVs) and intraclass correlation coefficients (ICCs) are also available in Murphy et al. (24).

**Table 1:** Lower limits of detection (LLOD) for human milk analytes from Murphy et al. (24).

Analytes	Analyte symbol	LLOD (pg/mL)
Interleukin-6	IL-6	0.14
Interleukin-8	IL-8	0.12
Tumor necrosis factor α	TNFα	0.09
Interleukin-1β	IL-1β	0.07
*Fas* ligand	FASL	0.415
Vascular endothelial factor D	VEGFD	3.26
Fms-related tyrosine kinase 1	FLT1	1.53
Placental growth factor	PLGF	0.42
Basic fibroblast growth factor	bFGF	0.15
Epidermal growth factor	EGF	0.075
Leptin	Leptin	93.96
Adiponectin	Adiponectin	0.08

Mean concentrations of human milk analytes were calculated for all 162 samples with duplicate values above the LLOD. The percent of mean values below the LLOD was calculated for each analyte. Values below the LLOD were imputed with the LLOD/2. Per Keizer et al. (26), single imputation with LLOD/2 has equivalent performance to maximum likelihood imputation when less than 10% of the sample is missing. For the 162 participants included in the current study, the majority of analytes (8 of 12) had less than 10% of samples below the LLOD. However, IL-6, IL-1β, TNFα, and bFGF ranged from 13 to 20% of values below the LLOD.

### 2.4 Statistical analysis

Data were cleaned and coded. All analyses were performed in SPSS 28.0. Descriptive statistics were examined. Spearman rank correlations and Mann–Whitney *U* tests were performed to explore bivariate associations. Two definitions of *elevated MEP* were examined in our dataset: Na ≥ 10 mmol/L (18, 21), and Na/K ratio ≥ 0.6 (18). Multivariable logistic regression models were fitted to predict increased MEP from maternal–infant factors. Principal component analysis with varimax rotation was used to identify affinities between pro-inflammatory cytokines, growth factors, and adipokines after normalizing (natural log) and centering analytes. Based on sample size, 0.45 was designated as the threshold for factor loadings (27). Factor scores were computed based on these results. Multivariable logistic regression models were fitted to examine factor scores as predictors of elevated MEP.

## 3 Results

### 3.1 Descriptive statistics for Na and the Na/K ratio

**Figure 1:**
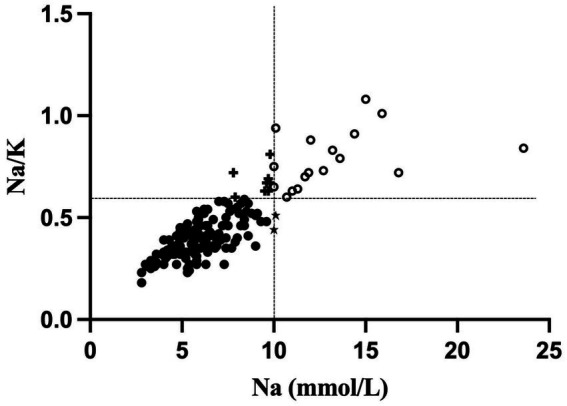
Scatterplot of sodium (Na; mmol/L) and the sodium-potassium (Na/K) ratio. Elevated MEP cases identified only by a Na level ≥ 10 mmol/L are indicated with a star (*n* = 2). Elevated MEP cases identified only by a Na/K ratio ≥ 0.6 are indicated with a plus sign (*n* = 8). Elevated MEP cases identified by both criteria are located in the right upper quadrant and are shown as open circles.

Descriptive statistics for Na, K, and Na/K ratios are presented in Table 2. Median concentrations of Na and K were 6.2 mmol/L (range 2.8–23.6) and 14.6 mmol/L (range 10.1–28.1) respectively. The median Na/K ratio in this sample was 0.41 with a range of 0.18–1.08. Na and the Na/K ratio were strongly correlated, *r*(160) = 0.79, *p* < 0.001. Two sets of criteria for identifying elevated MEP were examined. Using the criteria defined in the literature of 10 mmol/L for Na (18, 21), the cutoff for elevated MEP was at the 90th percentile and a total of 19 cases of elevated MEP were identified. Using the criteria of 0.6 for the Na/K ratio (18), the cutoff for elevated MEP was at the 85th percentile and 25 cases were identified. A total of 17 cases were identified by both criteria. Two cases were identified by Na ≥ 10 mmol/L alone, while 8 cases were identified solely by Na/K ≥ 0.6 (Figure 1).

**Table 2:** Descriptive statistics for Na, K, and the Na/K ratio (*N* = 162) and criteria for defining elevated MEP (cutoffs bolded) (18).

	Median	IQR	Elevated MEP criteria
Sodium (mmol/L)	6.2	5.0–8.0	**≥10 mmol/L** 90th percentile *N* = 19
Potassium (mmol/L)	14.6	12.7–17.4	N/A
Sodium-Potassium Ratio (Na/K)	0.41	0.34–0.52	**≥0.60** 85th percentile *N* = 25

### 3.2 Associations of MEP with mother-infant characteristics

Demographic and selected health characteristics of the 162 participants for the whole sample and by participant race are presented in Table 3. There was a weak negative association between maternal age and Na, *r*(160) = −0.19, *p* < 0.05, and the Na/K ratio, *r*(160) = −0.18, *p* < 0.05. Human milk Na levels also declined with older infant age, *r*(160) = −0.51, *p* < 0.001, but this association was not observed for the Na/K ratio. The majority of cases of elevated MEP occured in infants less than 4 months of age: 68% of cases identified by Na ≥ 10 mmol/L and 52% by Na/K ≥ 0.6.

**Table 3:** Median (IQR) or *N* (%) for maternal–infant characteristics for the entire sample (*N* = 162) and by participant race.

	*N*	Whole sample	*N*	Black	*N*	White	*p*-value
Maternal Age (Years)	162	31.0 (27.8–35.0)	98	31.0 (27.8–34.0)	64	32.0 (27.3–36.0)	0.21
Parity (Multipara)	162	88 (54%)	98	52 (53%)	64	36 (56%)	0.69
BMI (kg/m^2^)	162	25.9 (22.1–30.0)	98	26.3 (22.4–30.4)	64	24.6 (21.7–29.6)	0.11
Menses Since Birth (yes)	159	61 (38%)	97	43 (44%)	62	18 (29%)	0.06
Hours Since Pumping	123	2.0 (1.0–4.0)	87	2.0 (1.0–4.0)	36	3.0 (1.0–4.9)	0.15
Infant Age (weeks)	162	21.5 (11.8–38.7)	98	25.7 (13.1–44.4)	64	17.6 (10.6–30.0)	0.07

Maternal and infant characteristics were evaluated in multivariable logistic regression models predicting elevated MEP. Infant age and maternal race were significant predictors in the model predicting elevated MEP as indicated by Na ≥ 10 mmol/L (Table 4). For every additional week of infant age, the odds of elevated MEP as defined by Na declined by 4%. Black participants had a higher likelihood of experiencing elevated MEP by 3.3 times. BMI over 30 was not associated with elevated MEP as indicated by Na ≥ 10 mmol/L. No maternal or infant characteristics, including race or BMI, were significant in the model predicting elevated MEP as defined by the Na/K ratio ≥ 0.6. Medication use, shipping method, nor storage conditions were significant predictors of elevated MEP as indicated by Na or Na/K.

**Table 4:** Multivariable logistic regression model predicting elevated mammary epithelium permeability (MEP) as indicated by Na ≥ 10 mm/L (*n* = 162).

	OR (95% CI)
Infant Age (weeks)	**0.96 (0.93–0.99)**
Race (Black)^a^	**3.35 (1.03–10.82)**

Model 1: ^a^Reference: White.

Bolded values indicate a significant finding.

### 3.3 Human milk cytokines and associations with MEP

Descriptive statistics for the human milk cytokines are presented in Table 5. Both Na and the Na/K ratio had several significant positive associations with the selected cytokines. Nine analytes each were positively correlated with Na or the Na/K ratio, although the specific analytes varied by MEP indicator (see Table 5). Only leptin was not associated with either Na or the Na/K ratio. The overall pattern suggested that Na was more strongly associated with growth factors, while the Na/K ratio was more strongly associated with pro-inflammatory cytokines.

**Table 5:** Descriptive statistics for human milk analytes and Spearman rank correlations with Na and the Na/K ratio.

	*N*	Median (IQR)	Na	Na/K ratio
IL-6	162	1.53 (0.45–3.74)	0.35***	0.36***
IL-8	157	249.93 (132.46–629.40)	0.13	0.31***
TNFα	162	0.95 (0.46–2.51)	0.33***	0.28***
IL-1β	162	0.70 (0.27–1.81)	0.15	0.28***
FASL	162	43.23 (34.17–57.15)	0.28***	0.18*
VEGFD	161	275.92 (209.91–409.83)	0.25**	0.11
FLT1	161	1671.69 (1363.21–2423.49)	0.24**	0.12
PLGF	161	72.30 (41.77–127.57)	0.24**	0.26***
bFGF	162	0.98 (0.57–1.76)	0.41***	0.33***
EGF	162	4294.31 (3010.36–6052.75)	0.20*	0.22**
Leptin	162	1056.22 (490.75–2190.75)	0.11	0.07
Adiponectin	162	20.80 (15.70–28.46)	0.28***	0.17*

*p < 0.05, **p < 0.01, ***p < 0.001.

Principal components analysis, performed to consolidate the analytes, identified three unique factors (see Table 6). IL-6, IL-8, TNFα, IL-1β, and EGF loaded onto a factor representing the pro-inflammatory signature. The second factor was composed primarily of analytes involved in tissue growth and remodeling and included FASL, VEGFD, FLT1, PLGF, bFGF, and adiponectin. A single analyte, leptin, loaded onto the third factor. Pro-inflammatory and tissue remodeling factor scores were calculated based on this analysis.

**Table 6:** Analyte factor loadings for principal component analysis with varimax rotation.

	Factor 1 Pro-inflammatory	Factor 2 Tissue remodeling	Factor 3 Leptin
IL-6	**0.840**	0.188	0.201
IL-8	**0.849**	0.202	−0.078
TNFα	**0.829**	0.095	0.225
IL-1β	**0.843**	0.031	−0.017
FASL	0.212	**0.570**	0.315
VEGFD	−0.051	**0.871**	−0.070
FLT1	0.091	**0.816**	0.123
PLGF	0.216	**0.728**	−0.045
bFGF	0.393	**0.452**	0.388
EGF	**0.460**	0.423	−0.024
Leptin	0.054	−0.007	**0.892**
Adiponectin	0.395	**0.480**	−0.277

Factor loadings over the threshold of 0.45 are bolded.

Pro-inflammatory factor scores were higher in primipara as compared to multipara, Mann–Whitney *U* = 2,391, *p* < 0.05. The pro-inflammatory factor was more strongly correlated with Na/K, *r*(154) = 0.31, *p* < 0.001, than with Na alone, *r*(154) = 0.22, *p* < 0.01. In contrast, the tissue remodeling factor was more strongly correlated with Na, *r*(154) = 0.32, *p* < 0.001, than with the Na/K ratio, *r*(154) = 0.19, *p* < 0.05.

Multivariable logistic regression models were examined to evaluate pro-inflammatory and tissue remodeling factor scores as predictors of elevated MEP (Table 7). Controlling for infant age and maternal race, higher pro-inflammatory and tissue remodeling factor scores each uniquely raised the odds of elevated MEP as indicated by Na ≥ 10 mmol/L. A similar pattern was observed for the Na/K ratio where pro-inflammatory and tissue remodeling factors were uniquely associated with elevated MEP during established lactation.

**Table 7:** Multivariable logistic regression models and 95% confidence intervals predicting increased mammary epithelium permeability (MEP) from pro-inflammatory and tissue remodeling factor scores.

	OR (95% CI)
*Elevated MEP Na ≥ 10 mmol/L (n = 156)*	
Infant Age (weeks)	0.97 (0.93–1.00)
Race (Black)^a^	3.90 (0.98–15.54)
Pro-inflammatory Factor	**2.40 (1.39–4.17)**
Tissue Remodeling Factor	**2.55 (1.42–4.57)**
*Elevated MEP Na/K ≥ 0.6 (n = 156)*	
Pro-inflammatory Factor	**2.45 (1.51–4.00)**
Tissue Remodeling Factor	**2.02 (1.25–3.26)**

Model 1: ^a^Reference: White.

Bolded values indicate significant associations.

## 4 Discussion

To our knowledge, this is the first study to examine associations between MEP and cytokine profiles in human milk during *established lactation* among parents living in the US. Of note, the percentage of participants with elevated MEP was higher in this cohort than was observed in a European cohort during established lactation using the same criteria (Na/K > 0.6) (28). Elevated MEP during established lactation occurred in 5% or less of participants in a European cohort as compared to the observed 15% in the current study. However, worldwide prevelance of elevated MEP varies significantly (28, 29). More study will be needed to identify appropriate thresholds for different sub-populations.

Historically, MEP has been studied primarily in relation to secretory activation, subclinical mastitis (SCM), mastitis, and involution. Secretory activation occurs in the first days postpartum and is associated with a precipitous decline in MEP (18, 21), while SCM and mastitis may occur at any time during lactation and are associated with significant increases in MEP (2, 28, 30–32). Therefore, MEP has been linked to developmental processes, in addition to infectious processes such as subclinical mastitis or mastitis. Our aim is to understand the physiological process of MEP which can arise as the result of multiple lactation-related states.

The milk samples assessed in the present study were all from participants nursing infants between 1 and 18 months of age, and to our knowledge, no participant was experiencing symptoms of mastitis at the time of milk collection. While we did not specifically inquire about plans for weaning, it is possible that some participants may have been in the midst of this process. MEP, as indicated by elevated Na or Na/K, is known to increase during involution (33). Of note, the odds of elevated MEP (defined as Na ≥ 10 mmol/L) declined with advancing infant age overall, possibly representing normal changes in mammary function over the course of the lactation cycle. This is in line with previous research showing a decline in subclinical mastitis with older infant age (28, 30). However, few studies have examined these trends into the second year postpartum.

As predicted, Black participants in our cohort were more likely to experience elevated MEP (defined as Na ≥ 10 mmol/L), despite a non-significant older infant age in this sub-group. Since this was the first study to assess MEP in a sizable (*n* = 98) sample of Black females in the US, it is unknown whether the 3.3 times greater odds of elevated MEP among Black participants was due to socioeconomic conditions impacting the frequency of breastfeeding or pumping, represents normal variation in healthy breast tissue, or was a sign of inflammation in the mammary gland. Elevated MEP among Black participants is consistent with our previous report demonstrating higher levels of some pro-inflammatory cytokines in the milk of Black women in this cohort (24).

Levels of all cytokines examined were positively associated with either Na or the Na/K ratio, with the exception of leptin. A body of research has previously identified links between cytokines and permeability. Many of these studies have suggested that inflammation may drive increased permeability (2, 4, 31, 32). Indeed, during mastitis open paracellular pathways may be adaptive, allowing cytokine-producing leukocytes access to the alveolar lumen (34). However, permeability may also increase in response to the rising alveolar pressure associated with milk accumulation. Eventually, this can lead to tissue remodeling as seen during involution. Both pro-inflammatory cytokines and tissue growth factors play a role in this process (14, 19).

In the current study, pro-inflammatory and tissue remodeling factors each uniquely raised the odds of experiencing elevated MEP as indicated by either Na ≥ 10 mmol/L or the Na/K ratio ≥ 0.6. However, when considering continuous measures of Na or the Na/K ratio, a pattern emerged where the pro-inflammatory factor was more strongly associated with the Na/K ratio, while the tissue remodeling factor was more tightly linked with Na. To our knowledge, this is the first study to examine growth factors in relation to MEP. While additional research is needed to confirm this finding, unique patterns of Na and Na/K with specific cytokines could be used to identify the physiologic processes underlying elevated MEP.

Identifying the etiology underlying MEP has important implications for both parent and infant. Elevated MEP has been linked to delayed onset of lactation (18), low milk supply (35), reduced milk nutrient content (28), and inadequate infant growth (4). Infant growth faltering has also been identified in relation to elevated cytokines in human milk (3). Growth factors in human milk may also affect the development of the infant intestinal barrier (11). Identifying persistently increased permeability could also have important implications for identifying breast cancer risk, given the established role of cytokines in tumorigenesis (9, 24, 36). Taken together, both MEP and cytokines could have important implications for both parent and infant health.

An important direction for future research is to determine how to most appropriately measure MEP. In the present study, analyses based on Na levels and Na/K ratios provide slightly different results. Using cut-off values from the literature (18), Na was the more conservative indicator of elevated MEP in this study, identifying 19 cases as compared to the 25 cases identified using the Na/K ratio. Of note, a subset of 17 cases was identified by both indicators, while 2 cases were identified by Na alone, and 8 cases by the Na/K ratio alone. As noted above, continuous measures of Na were also more closely associated with tissue growth and remodeling cytokines, while the Na/K ratio seemed to be more tightly linked with pro-inflammatory cytokines. Additional research is needed to determine how best to interpret human milk Na levels and Na/K ratios during established lactation.

There were several strengths to the current study including a cohort with a significant number of Black female participants, representation of parents with up to 18 months of established lactation, and a panel of 12 cytokines. However, there is also an important limitation. This secondary analysis used milk and questionnaire data from a study aimed at understanding factors associated with breast cancer risk. Therefore, the study design and questionnaires were not optimally structured to assess factors known to be associated with MEP. In addition, more research is needed to determine if extended freezer storage at −20°C might affect the measurement of Na by ion selective electrode.

## 5 Conclusion

Results presented here highlight the importance of measuring mammary epithelium permeability in studies of normal developmental and inflammatory processes in the human mammary gland, as well as in studies of the effects of human milk on infant health. Surprisingly, the human mammary gland is the only organ for which we lack routine clinical tests for normal function (37, 38). Rich information may be obtained through the measurement of MEP indicators during established lactation. Research aimed at understanding the importance of MEP for mammary gland health or infant growth and development should be prioritized.

## Data availability statement

The raw data supporting the conclusions of this article will be made available by the authors, without undue reservation.

## Ethics statement

The studies involving humans were approved by University of Massachusetts Amherst Institutional Review Board. The studies were conducted in accordance with the local legislation and institutional requirements. The participants provided their written informed consent to participate in this study.

## Author contributions

KK: Conceptualization, Formal analysis, Writing – original draft. SS: Conceptualization, Methodology, Writing – review & editing. EB: Data curation, Investigation, Methodology, Validation, Writing – review & editing. BP: Writing – review & editing. DA: Writing – review & editing. KA: Conceptualization, Investigation, Methodology, Resources, Validation, Visualization, Writing – review & editing.

## References

[1] Say B Dizdar EA Degirmencioglu H Uras N Sari FN Oguz S. The effect of lactational mastitis on the macronutrient content of breast milk. Early Hum Dev. (2016) 98:7–9. doi: 10.1016/j.earlhumdev.2016.03.009, PMID: 27341630

[2] Li C Solomons NW Scott ME Koski KG. Subclinical mastitis (SCM) and proinflammatory cytokines are associated with mineral and trace element concentrations in human breast milk. J Trace Elem Med Biol. (2018) 46:55–61. doi: 10.1016/j.jtemb.2017.11.010 29413111

[3] Saso A Blyuss O Munblit D Faal A Moore SE Le Doare K. Breast Milk cytokines and early growth in Gambian infants. Front Pediatr. (2018) 6:414. doi: 10.3389/fped.2018.00414, PMID: 30705878 PMC6344434

[4] Li C Solomons NW Scott ME Koski KG. Anthropometry before day 46 and growth velocity before 6 months of Guatemalan breastfed infants are associated with subclinical mastitis and milk cytokines, minerals, and trace elements. J Nutr. (2019) 149:1651–9. doi: 10.1093/jn/nxz109, PMID: 31187864

[5] Brenmoehl J Ohde D Wirthgen E Hoeflich A. Cytokines in milk and the role of TGF-beta. Best Pract Res Clin Endocrinol Metab. (2018) 32:47–56. doi: 10.1016/j.beem.2018.01.006 29549959

[6] Palmeira P Carneiro-Sampaio M. Immunology of breast milk. Rev Assoc Medica Bras. (2016) 62:584–93. doi: 10.1590/1806-9282.62.06.584 27849237

[7] Porcherie A Gilbert FB Germon P Cunha P Trotereau A Rossignol C. IL-17A is an important effector of the immune response of the mammary gland to *Escherichia coli* infection. J Immunol Baltim Md. (2016) 196:803–12. doi: 10.4049/jimmunol.1500705 26685206

[8] Hunt KM Williams JE Shafii B Hunt MK Behre R Ting R. Mastitis is associated with increased free fatty acids, somatic cell count, and interleukin-8 concentrations in human milk. Breastfeed Med Off J Acad Breastfeed Med. (2013) 8:105–10. doi: 10.1089/bfm.2011.0141, PMID: 22283504 PMC3568962

[9] Arcaro KF Browne EP Qin W Zhang K Anderton DL Sauter ER. Differential expression of cancer-related proteins in paired breast milk samples from women with breast cancer. J Hum Lact. (2012) 28:543–6. doi: 10.1177/0890334412453205, PMID: 22914689

[10] Qin W Zhang K Kliethermes B Ruhlen RL Browne EP Arcaro KF. Differential expression of cancer associated proteins in breast milk based on age at first full term pregnancy. BMC Cancer. (2012) 12:100. doi: 10.1186/1471-2407-12-100, PMID: 22436421 PMC3412716

[11] Ballard O Morrow AL. Human milk composition: nutrients and bioactive factors. Pediatr Clin N Am. (2013) 60:49–74. doi: 10.1016/j.pcl.2012.10.002, PMID: 23178060 PMC3586783

[12] Kiełbasa A Gadzała-Kopciuch R Buszewski B. Cytokines-biogenesis and their role in human breast milk and determination. Int J Mol Sci. (2021) 22:6238. doi: 10.3390/ijms22126238, PMID: 34207900 PMC8229712

[13] Watson CJ Oliver CH Khaled WT. Cytokine signalling in mammary gland development. J Reprod Immunol. (2011) 88:124–9. doi: 10.1016/j.jri.2010.11.006 21255846

[14] Jena MK Jaswal S Kumar S Mohanty AK. Molecular mechanism of mammary gland involution: an update. Dev Biol. (2019) 445:145–55. doi: 10.1016/j.ydbio.2018.11.002, PMID: 30448440

[15] Colleluori G Perugini J Barbatelli G Cinti S. Mammary gland adipocytes in lactation cycle, obesity and breast cancer. Rev Endocr Metab Disord. (2021) 22:241–55. doi: 10.1007/s11154-021-09633-5, PMID: 33751362 PMC8087566

[16] Lamote I Meyer E Massart-Leën AM Burvenich C. Sex steroids and growth factors in the regulation of mammary gland proliferation, differentiation, and involution. Steroids. (2004) 69:145–59. doi: 10.1016/j.steroids.2003.12.008, PMID: 15072917

[17] Savilahti E Saarinen KM. Colostrum TGF-beta-1 associates with the duration of breast-feeding. Eur J Nutr. (2007) 46:238–42. doi: 10.1007/s00394-007-0656-9, PMID: 17497075

[18] Medina Poeliniz C Engstrom JL Hoban R Patel AL Meier P. Measures of secretory activation for research and practice: an integrative review. Breastfeed Med. (2020) 15:191–212. doi: 10.1089/bfm.2019.0247 32155345

[19] Stelwagen K Singh K. The role of tight junctions in mammary gland function. J Mammary Gland Biol Neoplasia. (2014) 19:131–8. doi: 10.1007/s10911-013-9309-1 24249583

[20] Nguyen DA Parlow AF Neville MC. Hormonal regulation of tight junction closure in the mouse mammary epithelium during the transition from pregnancy to lactation. J Endocrinol. (2001) 170:347–56. doi: 10.1677/joe.0.1700347, PMID: 11479131

[21] Neville MC Keller RP Seacat J Casey CE Allen JC Archer P. Studies on human lactation. I. Within-feed and between-breast variation in selected components of human milk. Am J Clin Nutr. (1984) 40:635–46. doi: 10.1093/ajcn/40.3.635 6475828

[22] Lai CT Gardner H Geddes D. Comparison of inductively coupled plasma optical emission spectrometry with an ion selective electrode to determine sodium and potassium levels in human Milk. Nutrients. (2018) 10:E1218. doi: 10.3390/nu10091218 PMC6164336 30177589

[23] Stelwagen K Farr VC McFadden HA Prosser CG Davis SR. Time course of milk accumulation-induced opening of mammary tight junctions, and blood clearance of milk components. Am J Phys. (1997) 273:R379–86. doi: 10.1152/ajpregu.1997.273.1.R379, PMID: 9249575

[24] Murphy J Pfeiffer RM Lynn BCD Caballero AI Browne EP Punska EC. Pro-inflammatory cytokines and growth factors in human milk: an exploratory analysis of racial differences to inform breast cancer etiology. Breast Cancer Res Treat. (2018) 172:209–19. doi: 10.1007/s10549-018-4907-7, PMID: 30083950 PMC6191357

[25] Narayanaswamy V Pentecost BT Schoen CN Alfandari D Schneider SS Baker R. Neutralizing antibodies and cytokines in breast Milk after coronavirus disease 2019 (COVID-19) mRNA vaccination. Obstet Gynecol. (2022) 139:181–91. doi: 10.1097/AOG.0000000000004661, PMID: 35104067 PMC8759542

[26] Keizer RJ Jansen RS Rosing H Thijssen B Beijnen JH Schellens JHM. Incorporation of concentration data below the limit of quantification in population pharmacokinetic analyses. Pharmacol Res Perspect. (2015) 3:e00131. doi: 10.1002/prp2.131 26038706 PMC4448983

[27] Hair J Tatham R Anderson R Black W. Multivariate data analysis. 5th ed. London: Prentice Hall (1998).

[28] Samuel TM De Castro CA Dubascoux S Affolter M Giuffrida F Billeaud C. Subclinical mastitis in a European multicenter cohort: prevalence, impact on human Milk (HM) composition, and association with infant HM intake and growth. Nutrients. (2019) 12:105. doi: 10.3390/nu12010105, PMID: 31905959 PMC7019749

[29] Aryeetey RNO Marquis GS Timms L Lartey A Brakohiapa L. Subclinical mastitis is common among Ghanaian women lactating 3 to 4 months postpartum. J Hum Lact. (2008) 24:263–7. doi: 10.1177/0890334408316077, PMID: 18689713

[30] Pace RM Pace CDW Fehrenkamp BD Price WJ Lewis M Williams JE. Sodium and potassium concentrations and somatic cell count of human milk produced in the first six weeks postpartum and their suitability as biomarkers of clinical and subclinical mastitis. Nutrients. (2022) 14:4708. doi: 10.3390/nu14224708, PMID: 36432395 PMC9694808

[31] Schaub R Badiou S Viljoen J Dujols P Bolloré K Van de Perre P. The immune response to sub-clinical mastitis is impaired in HIV-infected women. J Transl Med. (2018) 16:296. doi: 10.1186/s12967-018-1667-4, PMID: 30359283 PMC6202806

[32] Tuaillon E Viljoen J Dujols P Cambonie G Rubbo PA Nagot N. Subclinical mastitis occurs frequently in association with dramatic changes in inflammatory/anti-inflammatory breast milk components. Pediatr Res. (2017) 81:556–64. doi: 10.1038/pr.2016.220, PMID: 27814344

[33] Kobayashi K Matsunaga K Tsugami Y Wakasa H Nishimura T. IL-1β is a key inflammatory cytokine that weakens lactation-specific tight junctions of mammary epithelial cells. Exp Cell Res. (2021) 409:112938. doi: 10.1016/j.yexcr.2021.112938, PMID: 34800541

[34] Lehmann M Wellnitz O Bruckmaier RM. Concomitant lipopolysaccharide-induced transfer of blood-derived components including immunoglobulins into milk. J Dairy Sci. (2013) 96:889–96. doi: 10.3168/jds.2012-5410 23219120

[35] Murase M Wagner EA Chantry JC Dewey KG Nommsen-Rivers LA. The relation between breast milk sodium to potassium ratio and maternal report of a milk supply concern. J Pediatr. (2017) 181:294–297.e3. doi: 10.1016/j.jpeds.2016.10.044, PMID: 27871690 PMC5274566

[36] King J Mir H Singh S. Association of cytokines and chemokines in pathogenesis of breast cancer. Prog Mol Biol Transl Sci. (2017) 151:113–36. doi: 10.1016/bs.pmbts.2017.07.003 29096891

[37] Boss M Gardner H Hartmann P. Normal human lactation: closing the gap. F1000Res. (2018) 7:F1000 Faculty Rev-801. doi: 10.12688/f1000research.14452.1 PMC6013763 29983914

[38] Hurst NM. Recognizing and treating delayed or failed lactogenesis II. J Midwifery Womens Health. (2007) 52:588–94. doi: 10.1016/j.jmwh.2007.05.005 17983996

