# Supplementary material for: Mammary epithelium permeability during established lactation: associations with cytokine levels in human milk
Source: Front Nutr. 2024 Feb 13;11:1258905. doi: 10.3389/fnut.2024.1258905 (PMC10900798; doi:10.3389/fnut.2024.1258905)
Supplement: Supplementary file 1 [file Table_1.pdf]

Table S1. Median (IQR) or *N* (%) for medication use, milk shipping methods, and storage conditions for the whole sample and by participant race.

	N	**Whole Sample**	*N*	**Black**	*N*	**White**	***p*-value**
Prescriptions (Yes)	161	43 (27%)	97	25 (26%)	64	18 (28%)	0.74
Over-the-counter pain medications (Yes)	162	58 (36%)	98	35 (36%)	64	23 (36%)	0.98
Samples Shipped (Yes)	162	112 (69%)	98	91 (93%)	64	21 (33%)	<0.001
Freeze-Thaw Cycles	143	2 (2-2)	97	2 (2-2)	46	2 (2-2)	0.10
Storage at -20°C (Years)	151	10 (9-12)	93	9 (9-10)	58	13 (10-14)	<0.001
